# Unexpectedly High Levels of Inverted Re-Insertions Using Paired sgRNAs for Genomic Deletions

**DOI:** 10.3390/mps3030053

**Published:** 2020-07-29

**Authors:** Joseph Blayney, Evangeline M. Foster, Marta Jagielowicz, Mira Kreuzer, Matteo Morotti, Katharina Reglinski, Julie Huiyuan Xiao, Philip Hublitz

**Affiliations:** 1 MRC Molecular Hematology Unit, MRC Weatherall Institute of Molecular Medicine, University of Oxford, John Radcliffe Hospital, Headley Way, Oxford OX3 9DS, UK; joseph.blayney@stx.ox.ac.uk; 2 Translational Neuroscience and Dementia Research Group, Department of Psychiatry, University of Oxford, Warneford Hospital, Warneford Lane, Oxford OX3 7JX, UK; evangeline.foster@bnc.ox.ac.uk; 3 MRC Human Immunology Unit, MRC Weatherall Institute of Molecular Medicine, University of Oxford, John Radcliffe Hospital, Headley Way, Oxford OX3 9DS, UK; marta.jagielowicz@ndm.ox.ac.uk (M.J.); katharina.reglinski@rdm.ox.ac.uk (K.R.); julie4348@hotmail.com (J.H.X.); 4 Department of Oncology, MRC Weatherall Institute of Molecular Medicine, University of Oxford, John Radcliffe Hospital, Headley Way, Oxford OX3 9DS, UK; mira.kreuzer@oncology.ox.ac.uk (M.K.); matteo.morotti@wrh.ox.ac.uk (M.M.); 5 Department of Oncology, University of Lausanne, Ludwig Cancer Research Centre, HiTIDE group, Rue du Bugnon 25A, CH-1005 Lausanne, Switzerland; 6 Leibniz-Institute of Photonic Technologies & Institute of Applied Optic and Biophysics, Friedrich-Schiller University Jena, Max-Wien-Platz 1, D-07743 Jena, Germany; 7 MRC Weatherall Institute of Molecular Medicine, Genome Engineering Facility, University of Oxford, John Radcliffe Hospital, Headley Way, Oxford OX3 9DS, UK

**Keywords:** CRISPR/Cas9, dual sgRNA, genomic knock-out, NHEJ, PCR screen, inverted re-insertion, hidden genotypes

## Abstract

Use of dual sgRNAs is a common CRISPR/Cas9-based strategy for the creation of genetic deletions. The ease of screening combined with a rather high rate of success makes this approach a reliable genome engineering procedure. Recently, a number of studies using CRISPR/Cas9 have revealed unwanted large-scale rearrangements, duplications, inversions or larger-than-expected deletions. Strict quality control measures are required to validate the model system, and this crucially depends on knowing which potential experimental outcomes to expect. Using the dual sgRNA deletion approach, our team discovered high levels of excision, inversion and re-insertion at the site of targeting. We detected those at a variety of genomic loci and in several immortalized cell lines, demonstrating that inverted re-insertions are a common by-product with an overall frequency between 3% and 20%. Our findings imply an inherent danger in the misinterpretation of screening data when using only a single PCR screening. While amplification of the region of interest might classify clones as wild type (WT) based on amplicon size, secondary analyses can discover heterozygous (HET) clones among presumptive WTs, and events deemed as HET clones could potentially be full KO. As such, screening for inverted re-insertions helps in decreasing the number of clones required to obtain a full KO. With this technical note, we want to raise awareness of this phenomenon and suggest implementing a standard secondary PCR while screening for deletions.

## 1. Introduction

**Figure 1:**
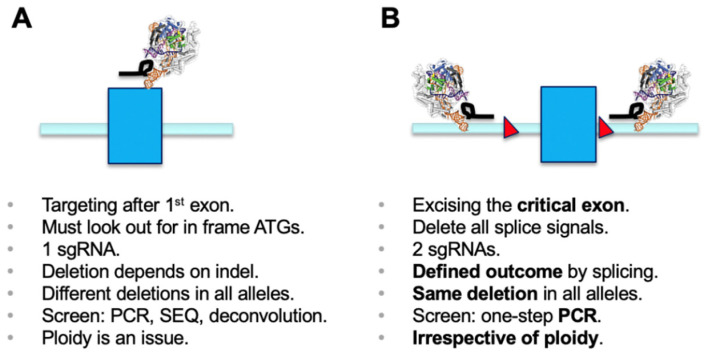
Two different approaches using CRISPR/Cas9 for achieving a functional KO, highlighting the differences that need to be considered. Exons are depicted as blue rectangles, splice branch and donor sites are indicated as red rectangles, and the crystal structure with the black loop indicates Cas9 and sgRNA. (**A**) Use of a single validated sgRNA relies on introducing a frameshift by NHEJ-mediated indel formation. When designing such guides, care must be taken to avoid later in-frame re-initiation of translation. Most alleles will have distinct deletion outcomes. Screening for mutants relies on PCR, sequencing and deconvolution of sequencing reads. More than diploid cell lines can be difficult to screen. The big advantage is ease (only one validated sgRNA is required) and least potential OFF-targeting. (**B**) Excision of a designated critical exon to generate a KO delivers a defined outcome of all clones that have been PCR screened for presence of the desired deletion. The same outcome will be obtained in all alleles due to exon ablation and subsequent aberrant splicing. The indel status in the intronic region generally does not contribute to any phenotype. A one-step PCR screen can identify mutations irrespective of ploidy.

Since the discovery of the CRISPR/Cas9 genome engineering technology platform [1], the most commonly used application has been generation of microdeletions using a single sgRNA. The cellular non-homologous end-joining (NHEJ) pathway repairs the double-strand breaks, and this can lead to generation of an out-of-frame gene knockout (KO) [2]. Functional outcomes of this process need to be properly screened for. Disruption of functional DNA motifs, such as transcription factor-binding sites or splicing signals, are easily achieved by deletion or insertion of one or more nucleotides. However, if a gene KO needs to be established, it is critical to make sure that a phase shift has been generated in all alleles of the target gene. It is worthwhile mentioning that using a single sgRNA has the inherent danger of generating different effects in sister alleles, which may result in distinct phenotypes. Screening usually involves a PCR over the region of interest, submitting the amplicon to sequencing, and deconvolution of the genotypes generated in all alleles (e.g., [2]) (Figure 1A).

Deletion of selected genetic elements relies on a dual sgRNA-mediated strategy and the repair follows the same principle as above, including excision of the genetic segment by Cas9 and subsequent repair of the cut by NHEJ [3,4]. For knockout of genes, as in classical gene targeting approaches, a chosen critical exon is deleted to generate a functional null allele [5]. The critical exon is defined by (i) being part of all transcriptional isoforms whose expression needs to be eliminated; (ii) being contained within the first 1/3 of the coding sequence to have a good chance for nonsense-mediated decay (NMD) to occur [6]; (iii) not being exon 1, if possible, and not having any immediate downstream in-frame ATGs (both of which could allow expression of a purely N-terminally truncated version); and (iv) having a base count indivisible by three to generate a phase shift in the transcript once the target exon is deleted. As such, deletion of the critical exon will create a defined mis-splicing and will terminate mRNA translation at a known endpoint. The targeting of intronic regions with sgRNAs needs to ensure all splicing signals are co-deleted, since only then will the previous exon be spliced to the subsequent downstream exon. A simple PCR screen over the target region discriminates presence and absence of the targeted exon/region and allows determination of deletion over WT alleles (Figure 1B) [4]. Presence of the short amplicon indicates deletion in all alleles analyzed and will identify the desired full KO. This is independent of ploidy and, in the primary screen, makes more complicated sequencing and deconvolution steps obsolete. Using the dual sgRNA approach is helpful when trying to establish deletions in triploid or tetraploid lines where generation of a full KO usually requires screening of many clonal lines. Several independent studies have reported the use of a dual sgRNA-based strategy being very effective, if not essential for genetic modification in diverse model systems [7,8,9,10,11].

CRISPR/Cas9 is widely used as a reliable and very precise genome engineering tool. However, accumulating evidence suggests that repair of Cas9-induced DNA double-strand breaks can lead to various degrees of genomic rearrangements. NHEJ is the default repair pathway for cells and is usually highly efficient and accurate to allow maintenance of cell viability [12], and inversions, duplications and deletions can be reliably generated by end joining after generation of DNA double-strand breaks [13,14]. However, when analyzing CRISPR/Cas9-mediated engineering with closer scrutiny, Shin and colleagues have identified large deletions of up to 600 bp and showed asymmetric deletions and large insertions of middle repetitive sequences [15]. Boroviak and colleagues demonstrated that larger genomic sequences targeted for inversion or excision can re-integrate (demarcated by the gRNAs) in the vicinity of the edited locus [16]. Furthermore, large deletions extending over many kilobases and more complex genomic rearrangements at the targeted sites were found, identifying significant numbers of unexpected cross-over events [17]. Our own research has observed frequent larger-than-expected deletions, potentially generated by the microhomology mediated end-joining (MMEJ) repair pathway [18]. Additionally, more recent findings report serial head-to-tail insertions of donor DNA templates [19]; error-prone repair pathways inserting unwanted deletions and insertions [20]; unintended ON-target chromosomal instabilities [21]; harmful chromosomal deletions [22]; and deleterious ON-target effects when aiming at homology directed repair (HDR) [23]. Hence, it is crucially important for precise model validation to know which outcomes CRISPR/Cas9 genome engineering can generate besides the actual aimed-for edit [24].

In addition to all of the above-described side products of genome editing, we observe an unexpectedly high level of inverted re-insertion events when using the dual sgRNA approach for genomic deletions. Our study summarizes several independent experiments, and we reveal inverted re-insertions in a median range of 3–20% throughout several different cell lines and at diverse genomic loci. Keeping this hitherto unreported phenomenon in mind, we suggest new measures that are vital for correct genotyping and, moreover, are potentially highly beneficial when establishing cellular model systems in more than diploid cell lines.

## 2. Materials and Methods

We present only a brief description of procedures and materials used in this manuscript since the aim is to provide a technical note as opposed to an elaborated protocol. However, we are very happy to share full details of experimental procedures, and any information request can be addressed to the corresponding author at any time.

Design of sgRNA reagents. All sgRNAs in this study were designed using the CRISPOR algorithm [25]. Guides aimed at direct excision of either the critical exon (within the first third of the coding sequence, number of base pairs not divisible by three to generate a phase shift after removal, present in all transcriptional isoforms) or the respective regulatory element as detailed in the text. Individual ssODNs for sgRNA pairs were subcloned into pX458-eGFP (Addgene 48138) and pX458-Ruby (Addgene 110164) and ON-target activity of each individual sgRNA was evaluated by Surveyor assays (according to the manufacturer, IDT). The two best and, as far as possible, equally well performing sgRNAs were chosen for the dual sgRNA-based deletions. All sgRNA pairs used for exon removal were designed to delete both the critical 5′ splice branching point and the 3′ splice donor sites to allow full removal of the critical exon. All sgRNAs used in this study target intronic or intergenic regions.

Cell lines. Cell lines were obtained from ATCC or SIGMA and cultured, as described (HeLa, 293, 293T-Rex, HT29-MTX-E12, HCT116, and 4T1). The hiPSC line CTR M3 36S was generated from the reprogramming of keratinocytes from a neurotypical Caucasian male, aged 36 years old, as described [26]. The mouse embryonic stem cell line E14 has been described [27].

Editing procedure. Two plasmids encoding one single sgRNA and color-coded Cas9 (eGFP for 5′ guides, and mRuby2 for 3′ guides) were transfected in either 6 or 12 well format into 293, HCT116, HeLa and 4T1 cells by lipofection with LPF_2000_ (ThermoFisher Scientific, Hemel Hempstead, UK ) and into HT29-MTX-E12 and E14 by Fugene (Roche). hiPSCs were transfected with pre-assembled RNPs using Alt-R^®^ S.p. HiFi-Cas9 nuclease 3NLS (IDT) and chemically synthesized tracrRNA and crRNA (IDT) using Lipofectamine CRISPRMAX™ reagent (ThermoFisher Scientific, Hemel Hempstead, UK), according to a previously outlined protocol [28]. All delivery procedures were used, as described by the respective manufacturers. Seventy-two hours post transfection, eGFP and mRuby2 double-positive cells were single-cell sorted by FACS and grown out to individual clonal lines. hiPSCs and E14 cells were plated in low density and subsequently picked and expanded as individual clonal lines.

Isolation of gDNA. gDNA was isolated by lysing the cell pellet of a confluent 6 or 12 well plate in 500 µL lysis buffer (50 mM Tris HCl pH8.0, 100 mM EDTA, 100 mM NaCl, 1% SDS, 100 µg proteinase K) by incubation overnight at 56 °C. gDNA was isopropanol-precipitated and (critical step) DNA pellets were resuspended overnight at 56 °C in 500 µL ddH_2_O before determination of the DNA concentration (Nanodrop, ThermoFisher Scientific, Hemel Hempstead, UK).

Screening and validation procedures. PCR screening procedures in this study involve the following setup: primers for amplification of the genomic fragments were designed as 23mers with the aim to have three 3′ G/C residues and a GC content of minimum 55%. Primary PCR amplification was performed using Qiagen Taq polymerase and standard buffers (QIAGEN, Hilden, Germany) in presence of 3% DMSO using an annealing temperature of 62 °C and an elongation time of 60 s for all amplicons below 1 kb using primers denoted FW (forward) and RV (reverse) (see Supplemental Table S1). PCR reactions were separated on 1% TAE agarose gels, pictures were archived and processed with a GelDoc XR gel documentation unit (BioRad, Hercules, USA). As soon as a WT band was amplified in conjunction with a deletion, samples were re-analyzed with co-aligned primers that will allow amplification only in case of inverted re-insertion occurred (oligonucleotides FW and FW*, described in Supplemental Table S1). Inversion events were confirmed by Sanger sequencing of the amplified WT-sized bands in both directions. Cell lines have been further characterized for either absence of protein expression by Western Blot, or for absence of mRNA expression by qRT-PCR.

All oligos used in this study are summarized in Supplementary Table S1.

## 3. Results

**Figure 2:**
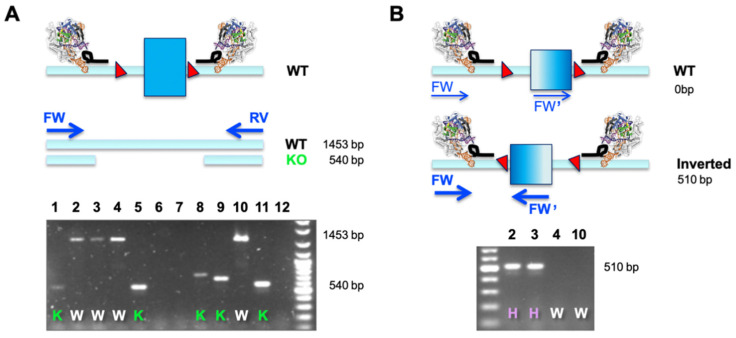
Typical PCR screening outcomes when performing a dual sgRNA-mediated deletion. Gel images are based on representative data from the R3 deletion generated in mouse E14 ESCs (Hba R3), as described in Table 1. (**A**) After isolation of clonal lines, gDNA is isolated and PCR primers flanking the critical exon are used to amplify either WT or deletion fragments. Presence of the shorter fragment is indicative of deletions, and this approach is irrespective of cellular ploidy. The agarose gel gives a representative result of 12 independently screened clones, with 5 full KOs (K; lines 1, 5, 8, 9 and 11) and 4 unedited clones (W; lines 2, 3, 4 and 10). Three clones did not yield any band, possibly due to low-quality gDNA. (**B**) We observe an unexpectedly high number of clones with excisions and inverted re-insertions that would appear as WT if uncontested (see also Table 1). Such events do qualify as bona fide null alleles due to the co-inverted splicing signatures and consequent exon skipping. We generally apply a secondary PCR screen using two FW oligos (FW and FW’ as indicated in the schematics) where only re-inserted inversion of the excised fragment will generate an amplicon. As such, from clones 2, 3, 4 and 10 (previously classified as WT as shown in Figure 2A), clones 2 and 3 turned out to be at least heterozygously deleted, indicated by “H”. For more than diploid lines, a third PCR will be required to determine the full allelic spectrum (e.g., FW inside and RV inside; data not shown). This is especially important if bona fide CRISPR/Cas9 treated WT clones are required for appropriate controls, or if low numbers of full KO clones are obtained and heterozygous clones can be re-screened as detailed and potentially more full KO clones can be identified.

We routinely use a dual sgRNA-mediated excision approach for both functional gene KO and excision of regulatory genetic elements. Using a dual sgRNA-based strategy has the potential to double the number of potential OFF-target modifications; however, using highly selected sgRNAs, we have successfully generated many model systems and have been unable to detect significant numbers of aberrantly generated models. This can potentially be attributed to the fact that we generally apply careful initial PCR screening procedures that always take into account potential larger-than-expected deletions [18]. The dual sgRNA-based excision works well in a wide selection of cell lines or genomic loci (for strategy see Figure 2, and for a summary of results see Table 1).

A dual sgRNA-based deletion of a critical exon leads to a defined outcome in all alleles and does not pose the risk of potential hypo- or hypermorphic outcomes when independent alleles are differentially modified. Importantly, the desired edit is easy to screen for using a simple overlapping PCR (as outlined in Figure 2A). This is especially useful when engineering cell lines that are more than diploid, which comprises most of the immortalized cancer cell lines available. A single PCR reaction will determine the allelic status at the same time (WT or KO), and this assay assumes that only clones with a ‘*deleted only band*’ are bona fide KO cell lines (Figure 2A). The observation of smaller bands of different sizes is indicative of excessive NHEJ taking place after Cas9-mediated excision.

When correlating phenotypes to results from such a simple primary PCR screen, we observed several clones that did not match the expected behavior. Clones potentially identified as HET (presence of a larger and a smaller band) did not express functional protein or detectable amounts of mRNA (data not shown). Investigating this matter by molecular cloning and Sanger sequencing, we found that a substantial number of clonal cell lines displayed inverted re-insertion of the excised fragment (Table 1). We set up a simple second PCR screen, making use of two primers in co-alignment. In this assay, both forward (FW and FW’) primers would fail to amplify in cases where the target element is retained in its WT orientation (generating only two non-exponential linear amplicons). It is only when an inverted re-insertion event occurs that the previously co-aligned primers face each other and generate a productive amplicon (Figure 2B). The example below demonstrates how a combination of our two-tier screening PCR revealed that clones previously classified as WT (clones 2 and 3) were actually HET, and only clones 4 and 10 were bona fide WT cells that went through the engineering procedure (Figure 2A,B). This is an important finding, especially when aiming to use WT-classified clones, which have seen identical genome engineering reagents and procedures, as the generally accepted best possible control.

We subsequently screened several ongoing projects with this new approach. We observed a median inverted re-insertion rate of 3–20% of all clones screened. We find varying efficiencies in different cell lines and diverse target loci in our set of 12 independent experiments; however, our data strongly suggest a common phenomenon. It is important to note that the median range does exclude sample sizes with very limited numbers of clones available (as marked by asterisks in Table 1). These cases yielded very high levels (50%) of inverted re-insertion; however, no clear deduction of generality is possible due to low sample numbers. We decided to include those numbers (Table 1) to give an as broad as possible overview, especially since they provide direct evidence of inverted re-insertions happening. Moreover, our data also demonstrate a high rate of efficiency in the generation of full KO in our experimental cohort, with a median range from 2 to 43%. The same cutoff for low-number projects has been applied (Table 1, %Δ).

Given the rather high occurrence of inverted re-insertions, it is important to stress the significance of this finding and the implications it can have on isolated clonal cell lines. Inverted re-insertions result in co-inversion of splicing signals and render them unrecognizable. Supporting this, we were unable to detect protein and mRNA expression in various inverted re-insertion models by RT-PCR, qRT-PCR and Western Blot analyses (data not shown). The consequence is that cells initially screened and identified as WT (by a single PCR) could well turn out to be heterozygous deletions. Importantly, this can be highly beneficial for the generation of gene deficient models in tri- or tetraploid cell lines. Generation of a full KO in those cells is usually difficult and requires screening of tens to hundreds of cell lines to find those with the required three or four simultaneous deletion events. In light of our observations, re-screening of HET clones (some to several alleles deleted and one potential WT remaining) could increase the pool of fully deleted clones by identification of inverted re-insertion events without the requirement to screen additional lines. In line with this, we observe that a large proportion of full KO models in tetraploid HeLa and 4T1 cell lines have been generated as combinations of KO and re-inserted inversions, at 30% and 100%, respectively (Table 1).

Interestingly, hiPSCs, which are notoriously hard to modify [29], did not display this outcome (Table 1, bottom row). We screened 80 clones and only one was isolated with a proper monoallelic deletion. Re-targeting with a different set of sgRNAs with the aim of achieving a biallelic deletion resulted in the screening of an additional 140 clones. In this case, no clones were identified to contain either further deletions or inverted re-insertions. This might indicate a difficult genetic context or may represent an inherent feature of repair pathways active in hiPSCs. Further work is required to address this since our observations are based on one target locus in one hiPSC line. We thought to include this example in our manuscript to raise awareness, especially, since very many individual clones have been screened (Table 1 and data not shown).

## 4. Discussion

We routinely use dual sgRNA approaches to generate deletions in genes or regulatory elements. We consistently find high levels of full deletions in all alleles across a variety of different cell lines, from different species and origins and targeting at several distinct loci (Table 1).

Generally, the regions of interest are short, with critical exons or the targeted regulatory elements usually spanning less than a couple of hundred base pairs. A screen for deletion events can be performed easily using a reliable PCR over the deleted region and this is, importantly, independent of cell ploidy. When analyzing inconsistent gene or protein expression data in genotyped cell clones, we realized that this approach produced unexpected inverted re-insertions at rather high frequencies next to common NHEJ events at either sgRNA target site. We observe a range of 3–20% of events where the target exon and associated splicing signals have been inverted, contributing to the frequencies of heterozygous clones and as well to functional null alleles. Likewise, we observed inversion of regulatory elements targeted using the same dual sgRNA strategy. Our selection of cell lines (Table 1) is not comprehensive. However, it allows us inference that inverted re-insertions are a rather common by-product of dual sgRNA-mediated genome engineering. Our observations are backed by Birling and colleagues who also detect variable inversion events performing mouse and rat oocyte injections using Cas9 mRNA and in vitro transcribed sgRNAs [14]. In general, more data need to be analyzed to demonstrate general effects of cell line origin, genomic location and dependency on other factors.

Our new data are in line with recent publications where genome engineering resulted in unexpected larger rearrangements [15,17,18,19,20,21,22,23]. Our findings support the observations of Boroviak and colleagues [16]; however, the high-frequency occurrence of inverted re-insertions using the dual sgRNA approach has not previously been described in this context. We think it is important to highlight the implications when establishing genome-engineered model systems. The frequencies we observe are variable, differ between cell lines and likely depend on intrinsic factors such as sgRNA quality, sgRNA activity or the underlying genomic context. We see a broad range from 0 up to 50% inversion frequency. The higher numbers (50% marked with * and ** in Table 1) are most likely overstated due to low sample numbers in re-screening experiments; however, this still stresses the point that inverted re-insertions are a common phenomenon. Inverted re-insertion events in our hands did not give rise to protein in any of the full KO cell lines, reflecting successful trigger of NMD and generation of functional bona fide null alleles (data not shown). Our data, accumulated in Table 1, could even be a general underrepresentation if larger-than-expected deletions were not picked up [18]. The limited amount of cell lines, target loci and delivery options does not allow a generalized conclusion; however, the fact we consistently detect inverted re-insertions, apparently irrespective of the experimental approach, is worthwhile to take into account when screening engineered cell lines. A more systematic screening needs to be undertaken to account for a better representation of the overall rates of inverted re-insertions.

An important lesson we learned is that inverted re-insertions must be considered when selecting appropriate control cell lines. The best control line is always the cells that underwent the same engineering pipeline and that turn out to be “WT” or “HET” in respect to the engineered target. If inverted re-insertions are not properly screened for, such events can be missed. Cells could wrongly be classified “WT” but instead be “HET”, resulting in a “hidden genotype” issue. It is vital to ensure bona fide KO cell lines are compared to the properly characterized control lines. Equally important, inverted re-insertions can be very beneficial when engineering polyploid cell lines. In several cases, we observed that HeLa and 4T1 tetraploid lines with a full KO genotype comprise a combination of deletion and inverted re-insertion alleles to generate the full KO (Table 1 and data not shown). An additional PCR screening step is enough to ensure a reliable primary identification of the deletion status in cell lines (Figure 2).

Albeit we saw a consistent rate of inverted re-insertion throughout several immortalized human and mouse cell lines or murine embryonic stem cells, we failed to detect any event in hiPSCs (Table 1 last row). Here, screening of initially 80 cells yielded only one HET deleted cell line with no indication of any inverted re-insertion. Re-targeting of the HET cell line and subsequent screening of >140 hiPSC clones did not result in identification of any full KO or inversion event. Addressing cell viability, it has been reported that a homozygous deletion of the target gene Clusterin is viable, at least in a mouse model [30]. Our data points are based on one experimental cell line only; however, we screened many colonies and wanted to include those data to raise awareness. This phenomenon may be locus specific; however, it might also be intrinsic to hiPSCs and the use of their respective repair pathways. It is interesting to note that the hiPSCs were the only cell line edited with RNPs instead of plasmid delivered Cas9. Future investigation using other loci and other established hiPSC lines may help bring clarity to this issue.

The potential danger of increased OFF-targeting and/or risking potentially higher levels of genomic rearrangements by using two instead of only one sgRNA needs to be carefully considered. In our experience, the benefits prevail, allowing an easy screen and, so far, not experiencing serious OFF-targeting issues. We generally generate three to five independent KO lines and cross-compare their phenotype to ensure that we have generated the model system we intended to create. To be fully aware of any other OFF-targeting events, a comprehensive genome wide analysis is highly recommended, such as targeted locus amplification [31] or unbiased next-generation whole-genome sequencing [32]. The frequency of apparent OFF-targeting events is in discussion, and several papers provide arguments for [33,34,35,36] or against [37] the use of a dual sgRNA-based approach for generation of non-clinical model systems. Ultimately, a careful decision needs to be made to choose between mono or dual sgRNA-based deletions. To our understanding, it depends on the balance between ease of model development, screening complexity and added quality control procedures. We generally recommend use of several lines for direct comparison as well as demonstrative final assays per cell line, such as qRT-PCR or Western Blot analyses, to confirm complete absence of mRNA or protein production.

## 5. Conclusions

Our technical note raises awareness of two points: (1) a dual sgRNA-mediated KO strategy is a highly efficient experimental approach to achieve precise deletions that are easy to screen for and, importantly, are irrespective of the ploidy of the cell line concerned; (2) inverted re-insertions occur in a median range of 3 to 20%. It is important to note that potential WT or HET clones required as control cell lines, if not properly screened for, could be wrongly classified and could result in skewed interpretation of scientific data. Additionally, inversions (when guides are properly designed) will equivalently contribute to the pool of functional null alleles and thus can help reduce the number of clones required for screening when generating full KO lines, especially in non-diploid cell systems. This technical note aims to serve as an “eye opener” for screening and to empower new opportunities in model generation.

## Figures and Tables

**Table 1:** Screening cell lines for targeted deletions.

Cell line	Target	Total	WT/HET	Δ	%Δ	INV	%INV	
**293**	Exon **PEX5**	5	4	**1**	**20**	**1**	**20**	
**293 T-Rex**	Exon **PEX5**	26	18	**8**	**31**	**5**	**19**	
**293**	Exon **PEX14**	2	1	**1**	**50**	**1**	**50**	*
**293 T-Rex**	Exon **PEX14**	34	28	**6**	**18**	**1**	**3**	
**4T1**	Exon **Car9**	6	3	**3**	**50**	**3**	**50**	**
**E14 mESC**	Enhancer **Hba R3**	28	16	**12**	**43**	**3**	**11**	
**E14 mESC**	Enhancer **Hba R4**	62	53	**9**	**15**	**2**	**3**	
**E14 mESC**	Enhancer **Hba Rm**	27	17	**10**	**26**	**5**	**19**	
**HeLa**	Exon **SCL38A2**	23	17	**6**	**26**	**2**	**9**	
**HeLa**	Exon **SMPD1**	150	147	**3**	**2**	**4**	**3**	
**HT29-mTX-E12**	Exon **WFDC2**	20	18	**2**	**10**	**2**	**10**	
**CTR M3 36S hiPSC**	Exon **CLU**	80	80	**0**	**0**	**0**	**0**	***

We observe a total of 3–20% inverted re-insertions in a number of different cell lines edited by dual sgRNA Cas9 targeting. Target genes (human full capital, mouse capital first letter) are classified as KO (exon derived) or as structural (functional deletion of an annotated enhancer). The table summarizes total number of clones screened (Total), amount of either wild-type or heterozygous clones obtained (WT/HET), amount of full deletions (Δ) as number or percentage and detected inverted re-insertions (INV) by number and total percentage. Initially, all clones were identified by Sanger sequencing. After identification of the inverted re-insertion phenomenon, clones were analyzed by PCR using flanking-opposed primers (primary screen) and re-tested with internal co-aligned primer pairs once a WT band was observed over the expected deletion (see Figure 2). Most of the homozygous deleted clones had one of the alleles inverted, and absence of mRNA or protein was confirmed. Nota bene: (*) For this experiment in 293 cells, only very low cell numbers were available and as such the value of 50% is potentially misleadingly high. (**) The initial screen in 4T1 cells produced three independent WT/HET clones that have been re-targeted. Only three out of six selected independent clones have been analyzed and demonstrated to be inverted re-insertions in a fully deleted setting. More clones have been frozen down and were not evaluated further. As such, also here the 50% might be misleadingly high. (***) For the hiPSC cell line included in the table, only one out of the initially 80 clones screened was identified as a bona fide WT/HET clone. Re-targeting and screening of >140 subclones did not detect any additional deletion or inversion.

## Supplementary Materials

The following are available online at https://www.mdpi.com/2409-9279/3/3/53/s1, Table S1: List of all oligonucleotides used in this study.
