# Supplementary material for: Unexpectedly High Levels of Inverted Re-Insertions Using Paired sgRNAs for Genomic Deletions
Source: Methods Protoc. 2020 Jul 29;3(3):53. doi: 10.3390/mps3030053 (PMC7565582; doi:10.3390/mps3030053)
Supplement: Supplementary file 1 [file mps-03-00053-s001.pdf]

Table S1: List of all oligonucleotides used in this study**.** All oligos are displayed in 5′ to 3′ orientation. Capital letters in the guide sequences highlight (a) BbsI adaptors (CACC) and (b) changes to G residues made to enable efficient pol III transcription.

TARGET	Type of Oligonucleotide	SEQUENCE
**Hba R3**	5' sgRNA with BbsI adaptor	CACC gggaagtgtaagtgttcag
	3' sgRNA with BbsI adaptor	CACC ggactggcagaaagctatgt
	Screen_FW	tgtgaaatcaccagaattacagg
	Screen_RV	tgtgggtcaggcctcttagaggg
	Screen_FW*	ccttctagaactagtcttatcac
**Hba R4**	5' sgRNA with BbsI adaptor	CACC Ggtggacaccttgggagttg
	3' sgRNA with BbsI adaptor	CACC GGctagctgaataatttgcgg
	Screen_FW	tttgccagcaaccttcacaggag
	Screen_RV	attttaaaggcattgcagagccg
	Screen_FW*	tgaacagacagacctgatctgag
**Hba Rm**	5' sgRNA with BbsI adaptor	CACC Ggaccagcgtagtctaactcc
	3' sgRNA with BbsI adaptor	CACC Ggactcaaccacatgactca
	Screen_FW	tagggaaaggttatgtgaactgc
	Screen_RV	tcaggctgggcactggctctgcc
	Screen_FW*	tgaattctctgaaaggtgaatcc
**WFDC2/HE4**	5' sgRNA with BbsI adaptor	CACC Ggtatcactcttggccccggt
	3' sgRNA with BbsI adaptor	CACC Ggctcggcccctctaggcgcc
	Screen_FW	tttaacccgcatgctctactgcc
	Screen_RV	gtcggggccctcagatctcagcc
	Screen_FW*	aatccccgcacctgagcatcggc
**Car9**	5' sgRNA with BbsI adaptor	CACC GGcgttaggataagtgagggg
	3' sgRNA with BbsI adaptor	CACC GGccctgctgaggtggggact
	Screen_FW	cacagtcattggagctatggagg
	Screen_RV	acgtgaatgtcaaatagggtacc
	Screen_FW*	cacgcagtacagctgactctgcc
**SLC38A2**	5' sgRNA with BbsI adaptor	CCAC Ggctaaagctgtaccaaagt
	3' sgRNA with BbsI adaptor	CCAC Ggagcctctcaccagttagt
	Screen_FW	cctggaataagtcctggtttggg
	Screen_RV	tgctgggaagactttaggcac
	Screen_FW*	ttagattgtggtatctgaacggg
**PEX5**	5' sgRNA with BbsI adaptor	CACC ggggtcgcagcaaaagcact
	3' sgRNA with BbsI adaptor	CACC Ggtttataaacgctcagtaag
	Screen_FW	ggtccaggcccctttgtggaggc
	Screen_RV	aacaagcaggcattctcattcgg
	Screen_FW*	aagcccaggtgcagcctctgaggc
**PEX14**	5' sgRNA with BbsI adaptor	CACC Ggatcagctcgaatggagatc
	3' sgRNA with BbsI adaptor	CACC GGacccccagtggggcatgc
	Screen_FW	acgtttagacaggtggaggcagg
	Screen_RV	tttgtcagggtccactttatccc
	Screen_FW*	gccagagcccacttgcaaccagg
**SMPD1**	5' sgRNA with BbsI adaptor	CACC GGtgagttacagggcaatatc
	3' sgRNA with BbsI adaptor	CACC Ggagccaaatgaagagcacta
	Screen_FW	tattcaccgccatcaacctcggg
	Screen_RV	ccagcattttgggaggccgaggc
	Screen_FW*	ggccctgaccaccgtcacagcac
**CLUSTERIN**	5' sgRNA with BbsI adaptor	CACC GGctgccttcgctttcattcg
	3' sgRNA with BbsI adaptor	CACC GGacactggggcctgatagag
	Screen_FW	gataccactctggacaaggaggc
	Screen_RV	cacaggagggataagctctgggc
	Screen_FW*	gtctcctgttctctgcagaggcg
